# Semaglutide in Metabolic Dysfunction-Associated Steatohepatitis: A Narrative Review

**DOI:** 10.7759/cureus.95632

**Published:** 2025-10-28

**Authors:** George S Zacharia, Sudharsan R Gongati, Aayush Kharel, Anu Jacob

**Affiliations:** 1 Internal Medicine, BronxCare Health System, New York City, USA; 2 Gastroenterology and Hepatology, Ahalia Hospital Mussafah, Abu Dhabi, ARE; 3 Anesthesiology and Perioperative Medicine, Ahalia Hospital Mussafah, Abu Dhabi, ARE

**Keywords:** glp-1 agonists, metabolic-dysfunction associated steatohepatitis (mash), obesity, semaglutide, type 2 diabetes mellitus (t2dm)

## Abstract

Glucagon-like peptide-1 (GLP-1), an incretin hormone, plays a crucial role in glucose homeostasis by stimulating insulin secretion, suppressing glucagon release, and delaying gastric emptying. Its therapeutic potential was long realized, leading to the development of the first GLP-1 receptor agonist, exenatide, followed by liraglutide, dulaglutide, semaglutide, and tirzepatide. Semaglutide is available as a weekly subcutaneous injection with high bioavailability. Semaglutide is the only GLP-1 agonist available for oral therapy, used in the treatment of type 2 diabetes mellitus (T2DM). Semaglutide has demonstrated broad clinical efficacy beyond glycemic control, including weight reduction, cardiovascular risk reduction, and, most recently, in the treatment of metabolic dysfunction-associated steatohepatitis (MASH). Semaglutide therapy is associated with the resolution of steatohepatitis and improvement in hepatic fibrosis in patients with MASH. Alongside resmetirom, semaglutide is currently approved for the treatment of non-cirrhotic MASH with moderate-to-advanced fibrosis. Safety considerations include gastrointestinal intolerance, hypoglycemia, rare pancreaticobiliary events, and theoretical concerns of thyroid C-cell tumors, though human risk remains minimal. In summary, semaglutide extends the armamentarium of the hepatologist against the most common liver disease worldwide.

## Introduction and background

Metabolic dysfunction-associated steatotic liver disease (MASLD) is the most common chronic liver disease worldwide, with an estimated prevalence of 1.27 billion across the globe in 2021 [1]. Its incidence has risen parallel to the global obesity pandemic. The age-standardized incidence rate of MASLD, per 100,000 population, increased from 475.54 in 1990 to 593.28 in 2021 and is expected to rise to 928.10 by 2045 [1]. Metabolic dysfunction-associated steatohepatitis (MASH) is a severe form of MASLD and has the potential to progress to cirrhosis and hepatocellular cancer. The burden of steatotic liver diseases calls for effective treatment options to mitigate the disease process. The diseases, though known for many decades, lacked an adequate, evidence-based therapy until 2024, when resmetirom was approved by the United States Food and Drug Administration (US FDA) [2]. Glucagon-like peptide-1 (GLP-1) and its agonists stimulate pancreatic insulin secretion owing to the incretin effect. Additionally, they suppress glucagon secretion, gastric emptying, and appetite, all contributing to weight loss and improved glycemic control. Most GLP-1 analogs have been evaluated in MAFLD/MASH with varying degrees of success, but fell short of approval until semaglutide, a long-acting GLP-1 agonist, was approved for treatment of MASH in 2025 [3].

This narrative review aims to comprehensively address the mechanism of action, scientific evidence, safety profile, and future directions of semaglutide specifically for the treatment of MASH. A comprehensive literature search was performed in PubMed/Medical Literature Analysis and Retrieval System Online (MEDLINE) to identify studies evaluating the role of semaglutide in steatotic liver diseases. The search strategy employed Boolean operators using the following terms: (“semaglutide” OR “GLP-1 receptor agonist”) AND (“MASLD” OR “MASH” OR “NASH” OR “NAFLD” OR “non alcoholic fatty liver disease” OR “non alcoholic steatohepatitis” OR “metabolic dysfunction associated steatotic liver diseases: OR “metabolic dysfunction associated steatohepatitis”). Only articles published in English since 2000 were included for review. Additional references were identified through manual review of relevant articles and reference lists.

## Review

GLP-1 agonists

GLP-1 was first identified in 1986, in the intestine, by Habener and Mojsov. The very next year, the insulinotropic effects of GLP-1 were demonstrated in rats, pigs, and human volunteers, substantiating its 'incretin' effect [4-6]. The native GLP-1 is secreted by the L cells of the distal jejunum and ileum. The secretion is primarily triggered by macronutrients in the lumen and, to a lesser extent, by neural signals and microbial products [7]. The physiological effects of GLP-1 include stimulation of pancreatic insulin secretion, attenuation of pancreatic glucagon secretion, delayed gastric emptying, and induction of satiety [8]. The GLP-1 in circulation has a very short half-life, typically a couple of minutes, as it is promptly degraded by the enzyme dipeptidyl peptidase-4 (DPP-4) [8].

In the early 1990s, several studies reported the role of these molecules in diabetic patients, with improvements in glycemic status and increased insulin levels following intravenous infusion of GLP-1 [9,10]. The subsequent heightened interest in a new therapeutic agent for DM culminated in the discovery of the first GLP-1 analogue, exenatide, which was approved by the US FDA for the treatment of type 2 diabetes mellitus (T2DM) in 2005 [11]. Liraglutide was approved for the treatment of T2DM in 2009 in the European Union and by the US FDA in 2010. Beyond its role in improving glycemic status, the drug was associated with significant weight loss of ≥5% in more than 50% of patients [12]. This finding broadened the focus on liraglutide from a pure diabetic medication to an agent of utility in obesity and metabolic syndrome. The SCALE (Satiety and Clinical Adiposity-Liraglutide Evidence trial) evaluated the role of liraglutide in participants with obesity, defined as a BMI ≥30 kg/m², or overweight individuals with a BMI ≥27 kg/m², along with comorbidities. The trial demonstrated a mean weight loss of 8.4 kg with once-daily, 3 mg subcutaneously administered liraglutide over 56 weeks, compared to 2.8 kg in the placebo arm [13]. Based on scientific evidence, liraglutide was approved by the US FDA for chronic weight management in 2014. The other available GLP-1 agonists include dulaglutide, semaglutide, and tirzepatide. Albiglutide and lixisenatide have been discontinued. Unlike other molecules, tirzepatide is a dual agonist, acting as both a GLP-1 receptor agonist and a glucose-dependent insulinotropic polypeptide (GIP) analog [14].

Semaglutide

**Figure 1:**
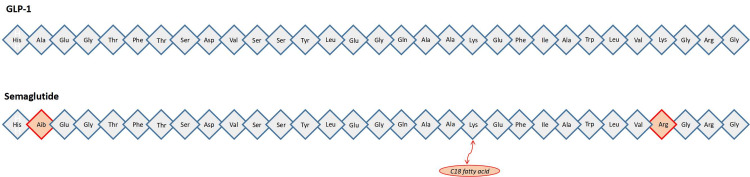
Protein sequence of native glucagon like peptide-1 (GLP-1)and semaglutide In semaglutide, the alanine residue at position 8 is replaced by α-aminoisobutyric acid, lysine is replaced by arginine at position 34, and a C18 fatty acid is conjugated to the lysine residue at position 26. This figure has been created by the authors.

Semaglutide is a GLP-1 analogue, where the alanine residue at position 8 is replaced by α-aminoisobutyric acid, a C18 fatty acid is conjugated to the lysine residue at position 26, and lysine is replaced by arginine at position 34 (Figure 1) [15]. These modifications make semaglutide less susceptible to dipeptidyl peptidase-4-mediated inactivation and enhance its binding to albumin, resulting in a prolonged half-life. Since its initial approval for T2DM, the benefits of the drug extend far beyond a mere antidiabetic agent, and it is currently approved for its beneficial effects on obesity/overweight, major adverse cardiovascular events, and MASH. In the United States, the drug is marketed under three distinct brands: Ozempic®, Rybelsus®, and Wegovy® [16]. Interestingly, each brand of semaglutide has a different dosage and indications (Table 1). 

**Table 1:** Currently marketed formulations of semaglutide US FDA: United States Food and Drug Administration; T2DM: type 2 diabetes mellitus; MASH: metabolic dysfunction-associated steatohepatitis

Semaglutide formulations					
Brand	US FDA Approval		Route of administration	Frequency	Dosing
	Indication	Year			
Ozempic®	T2DM	2017	Subcutaneous	Weekly	Prefilled pen: 2 mg, 4 mg, 8 mg
	Cardiovascular risk reduction in T2DM	2019			
	Reduced risk of worsening kidney disease, reduced risk of cardiovascular death	2019			
Rybelsus®	T2DM	2019	Oral	Daily	Tablet: 3 mg, 7 mg, 14 mg
Wegovy®	Obesity in adults	2021	Subcutaneous	Weekly	Prefilled pen: 0.25 mg, 0.5 mg, 1 mg, 1.7 mg, 2.4 mg
	Obesity in teens, aged ≥ 12 years	2022			
	Cardiovascular risk reduction in obesity	2024			
	MASH with modearte to advanced fibrosis	2025			

Semaglutide was approved by the US FDA in 2017 under the brand name Ozempic® for the management of T2DM. The PIONEER (Peptide InnOvatioN for Early diabEtes tReatment) trials confirmed the role of oral semaglutide in treating T2DM, leading to the US FDA approval of Rybelsus® in 2019 [17]. The SUSTAIN (Semaglutide Unabated Sustainability in Treatment of Type 2 Diabetes) trials, which used subcutaneous weekly semaglutide, demonstrated a reduction in major adverse cardiovascular events; however, the PIONEER trials failed to show the same benefit with oral semaglutide [17,18]. Yet another formulation of subcutaneous semaglutide, Wegovy®, demonstrated its efficacy in weight management in patients with obesity, specifically those with a BMI ≥30 kg/m² or those who were overweight (BMI ≥27 kg/m²) with comorbidities such as hypertension, T2DM, or dyslipidemia [19].

Semaglutide in MAFLD/MASH

MASLD/MASH, being closely associated with T2DM, metabolic syndrome, and obesity, and having had no effective treatment for decades, was increasingly considered yet another target by investigators. The Phase 2 trial by Newsome et al. evaluated weekly subcutaneous semaglutide at doses of 0.1 mg, 0.2 mg, and 0.4 mg compared to placebo in patients with biopsy-proven non-alcoholic steatohepatitis (NASH) and fibrosis stages F1 to F3. Statistically significant NASH resolution was observed in patients who received semaglutide, especially at a weekly dose of 0.4 mg, compared to placebo [20]. A 10-year retrospective Veterans Affairs study, published in 2024, reported significant improvements in transaminase levels and MASH scores with semaglutide [21]. The Phase 2 trial by Loomba et al., evaluating weekly 2.4 mg semaglutide, published in 2023, failed to achieve NASH resolution or improvement in fibrosis compared to placebo in patients with biopsy-proven NASH-related compensated cirrhosis [22]. The patient selection (subjects with established cirrhosis rather than those with advanced fibrosis without cirrhosis) might have yielded the negative result in this study. The Phase 3 ESSENCE (Effect of Semaglutide in Subjects with Non-cirrhotic Non-alcoholic Steatohepatitis) trial evaluated weekly 2.4 mg subcutaneous semaglutide in the treatment of biopsy-proven MASH with fibrosis F2 or F3, compared to placebo. The interim results published in April 2025 revealed a significant resolution of MASH in 62.3%, a reduction in fibrosis in 36.8%, and a combined resolution in steatohepatitis with improvement in fibrosis in 32.7% of subjects receiving semaglutide [23]. A large-scale, retrospective, real-world study by Suki et al. also reported improved overall and hepatic outcomes with semaglutide therapy in patients with MAFLD [24]. A pooled analysis of randomized controlled trials of semaglutide in MASH revealed a statistically significant p-value < 0.0001 for resolution of MASH and no worsening of fibrosis [25]. But the improvement in fibrosis without worsening of MASH failed to achieve statistical significance in the pooled analysis [25]. A retrospective trial by Kuo et al., including nearly 0.6 million patients with T2DM and non-alcoholic fatty liver disease (NAFLD), comparing semaglutide with other diabetic medications, revealed a lower incidence of major adverse hepatic outcomes and all-cause mortality in subjects on semaglutide [26]. Oral semaglutide was also associated with improvement in hepatic steatosis and markers of fibrosis in patients with T2DM and MASLD over a course of 48 weeks, according to a prospective study by Arai et al. [27]. Table 2 summarizes the recent trials of semaglutide in the management of MASLD/MASH [20-27].

**Table 2:** Summary of trials evaluating the role of semaglutide in MASLD/MASH ESSENCE: Effect of Semaglutide in Subjects with Non-cirrhotic Non-alcoholic Steatohepatitis; RCT: randomized controlled trial; MASH: metabolic dysfunction-associated steatohepatitis; CI: confidence interval; NASH: nonalcoholic steatohepatitis; MRE: magnetic resonance elastography; MRI-PDFF: magnetic resonance imaging-proton density fat fraction; ETR: estimated treatment ratio; NAFLD: nonalcoholic fatty liver disease; HCC: hepatocellular carcinoma; SGLT2i: sodium glucose cotransporter-2 inhibitors; DPP-4i: dipeptidyl peptidase-4 inhibitors; TZD: thiazolidinediones; MASLD: metabolic dysfunction–associated steatotic liver disease; ALT: alanine transaminase; AST: aspartate aminotransferase; FIB-4: fibrosis-4; CAP: controlled attenuation parameter; LSM: liver stiffness measurement; s/c: subcutaneous injection

Semaglutide Trials in MAFLD/MASH			
Trial; Type, Phase	Subjects	Intervention/Compatator	Outcomes
Newsome et al. [20] RCT, Phase II	Biopsy proven MASH; F1, F2 or F3	Intervention: semaglutide 0.1 or 0.2, or 0.4mg weekly s/c comparator: placebo	(a) NASH resolution with no worsening of fibrosis: semaglutide 40% (0.1 mg), 36% (0.2 mg), 59% (0.4 mg) vs. placebo 17% (P < 0.001 for 0.4 mg vs. placebo); (b) Improvement in fibrosis stage without worsening of NASH: semaglutide 43% (0.4 mg) vs. placebo 33% (P = 0.48); (c) Mean % change in body weight: semaglutide −5% (0.1 mg), −9% (0.2 mg), −13% (0.4 mg) vs. placebo −1%.
Loomba et al. [22] RCT; Phase II	Biopsy-confirmed NASH-related cirrhosis and BMI ≥27 kg/m2	Intervention: semaglutide 2.4mg weekly s/c comparator: placebo	(a) Improvement in liver fibrosis by ≥ 1 stage without worsening of NASH: Semaglutide 11% vs. Placebo 29% (odds ratio: 0.28; 95% CI 0.06 to 1.24; p=0.087); (b) NASH resolution: Semaglutide 34% vs. Placebo 21% (odds ratio: 1.97; 95% CI 0.56 to 7.91; p=0.29); (c) Liver stiffness measured using MRE (ratio to baseline): semaglutide 0.9 vs. placebo 0.97 (ETR: 0.93; 95% CI 0.8 to 1.07; p=0.3027); (d) Liver steatosis estimated with MRI-PDFF (ratio to baseline): semaglutide 0.68 vs. placebo 1.02 (ETR: 0.67; 95% CI 0.51 to 0.88; p=0.0042)
ESSENCE trial [23] RCT; Phase III	Biopsy proven MASH; F2 or F3	Intervention: semaglutide 2.4mg weekly s/c comparator: placebo	(a) Resolution of steatohepatitis without worsening of fibrosis: semaglutide 62.9% vs. placebo 34.3% (Δ 28.7%; 95% CI: 21.1 to 36.2; P < 0.001); (b) Reduction in liver fibrosis without worsening of steatohepatitis: semaglutide 36.8% vs. placebo 22.4% (Δ 14.4%; 95% CI: 7.5 to 21.3; P < 0.001); (c) Combined resolution of steatohepatitis and reduction in liver fibrosis: semaglutide 32.7% vs. placebo 16.1% (Δ 16.5%; 95% CI: 10.2 to 22.8; P <0.001); (d) Mean change in body weight: semaglutide -10.5% vs. placebo -2.0% (Δ -8.5%; 95% CI: -9.6 to -7.4; P < 0.001)
Kuo et al. [26] Retrospective cohort study	Adults with NAFLD and T2DM	Semaglutide versus SGLT2i, DPP-4i, TZD	(a) Major adverse liver outcomes: hepatic decompensation, HCC, or liver transplant Semaglutide vs. SGLT2i: adjusted hazard ratio = 0.73; 95% CI 0.60 to 0.88 Semaglutide vs. DPP-4i: adjusted hazard ratio = 0.72; 95% CI 0.56 to 0.86 Semaglutide vs. TZD: adjusted hazard ratio = 0.76; 95% CI 0.56 to 0.99 (b) All-cause mortality Semaglutide vs. SGLT2i: adjusted hazard ratio = 0.62; 95% CI 0.53 to 0.72 Semaglutide vs. DPP-4i: adjusted hazard ratio = 0.42; 95% CI 0.36 to 0.49 Semaglutide vs. TZD: adjusted hazard ratio = 0.67; 95% CI 0.54 to 0.83
Arai et al. [27] Prospective study	Adults with MASLD and T2DM	Oral semaglutide x 48 weeks, initiated at a dose of 3mg/day. No comparator	(a) ALT (IU/L): Baseline: 62 (43–89); 48 weeks: 35 (22–56) (p <0.001); (b) AST (IU/L): Baseline: 42 (32–61); 48 weeks: 27 (19–38) (p <0.001); (c) Type IV collagen (ng/mL): Baseline: 4.2 (3.5–5.1); 48 weeks: 3.7 (2.9–4.4) (p <0.001); (d) FIB-4 index: Baseline: 1.30 (0.84–2.25); 48 weeks: 1.09 (0.72–1.75) (p <0.001); (e) CAP (dB/m): Baseline: 318 (291–348); 48 weeks: 300 (263–327) (p <0.01); (f) LSM (kPa): Baseline: 7.1 (5.5–12.7); 48 weeks: 6.4 (4.6–8.9) (p <0.01)

The interim results of the ESSENCE trial led to the US FDA approval of Wegovy® for the treatment of non-cirrhotic MASH with moderate to advanced fibrosis (F2 or F3) in August 2025 [28]. Currently, weekly Wegovy® 2.4 mg, administered subcutaneously, is the only molecule approved for the treatment of MASH, apart from resmetirom. 

Semaglutide's Mechanism of Action

Due to its homology with GLP-1, it binds to and activates GLP-1 receptors. These receptors are most abundant in the pancreas and brain in humans. In the pancreas, GLP-1 receptor activation on β and δ cells stimulates insulin and somatostatin secretion, respectively; while acting on ɑ cells, it diminishes glucagon secretion. Somatostatin induces paracrine inhibition and reduces gut motility. GLP-1 receptor stimulation in the hypothalamus leads to anorexia and satiety. The activation of GLP-1 receptors on the gastrointestinal tract leads to reduced gastric smooth muscle contraction, food intake, and an enhanced incretin effect. The anorexia, satiety, and gastroparesis and gastric emptying [29,30]. Overall, GLP-1 receptor activation results in reduced appetite and contributes to weight loss, explaining the impact of semaglutide on obesity. Weight loss alleviates insulin resistance. The improved insulin sensitivity, combined with increased insulin secretion, prevents peripheral lipolysis, reduces free fatty acids in circulation and the liver, and decreases triglyceride synthesis and steatosis in hepatocytes. Reduced oral intake and gastroparesis curtail the luminal and portal venous fatty acids, which are believed to contribute to approximately 15% of hepatic triglycerides. Furthermore, semaglutide is postulated to inhibit hepatic de novo lipogenesis by interfering with ChREBP and SREBP-1c signaling pathways [30,31].

The exact mechanism of semaglutide in MAFLD/MASH is unclear; however, it is largely postulated to be a reflection of its anti-obesity and antidiabetic effects. The GLP-1 receptors are sparsely expressed on hepatocytes or hepatic stellate cells, again pointing against the possible direct effect of GLP-1 on the liver. Analysis by da Silva et al. reported that only <0.1% of liver cells express GLP-1 receptors, including isolated ones on the endothelium, stroma, NK/T cells, and hepatocytes [30,32]. Pleiotropic effects of semaglutide have been postulated as a mechanism in steatotic liver disease; however, it remains largely unproven. These include direct suppression of lipogenesis by downregulation of the lipogenic PI3K/AKT/mTORC1 signaling, activation of AMPK and SIRT1 responsible for fatty acid synthesis, and activation of PPAR-ɑ expression. Semaglutide is postulated to confer anti-inflammatory effects by downregulating prostaglandins, leukotrienes, galectin-3, TNF-α, IL-1β, and IL-6 [30,33]. Antifibrotic effects, mediated by the suppression of TGF-β1 expression, as well as antioxidant effects, are suggested based on preclinical studies [30,34]

Semaglutide Administration

Semaglutide preparations, such as Ozempic® and Wegovy®, are administered weekly as subcutaneous injections, typically over the abdomen, thighs, or upper arm [16]. Rybelsus® is administered orally, and as of mid-2025, it is the only GLP-1 agonist available for oral therapy. The oral tablet is administered once daily. A minimum 30-minute post-drug fasting and <120 ml of fluids for swallowing the drug is recommended for oral treatment [35]. Dosing of semaglutide preparations is summarized in Table 1. No dose adjustment is recommended in patients with hepatic or renal dysfunction [16,36]. Available literature is insufficient to assess the safety profile of semaglutide in pregnant and lactating females. Drummond et al. recommend contraception in all patients receiving GLP-1 agonists owing to the lack of evidence on their safety [37].

Semaglutide Pharmacokinetics

The subcutaneously administered semaglutide has nearly 90% bioavailability, while the oral preparations have poor bioavailability, about 0.8% under optimal conditions [16,35]. Interestingly, unlike most orally administered drugs, oral semaglutide is maximally absorbed from the stomach [38]. The oral absorption depends on the duration of the post-drug fasting time, the amount of fluid consumed with the drug, and the composition of the gastric fluid/content. A longer duration of post-drug fasting and lesser fluid intake with the drug accentuates the oral bioavailability [35].

The time to peak drug concentration (Tmax) with subcutaneously administered semaglutide is estimated to be 30 hours to 56 hours and 24 hours to 77.8 hours in healthy and diseased subjects, respectively. The Tmax of orally administered semaglutide is estimated to be 0.8 to 1.75 hours and 1.0 to 1.75 hours in healthy individuals and those with disease, respectively. The diseases that were taken into account include hepatic, renal, and upper gastrointestinal diseases [38]. The drug is metabolized by a combination of β-oxidation of the fatty acid side chain and proteolytic cleavage by DPP-4. The metabolism is not organ-specific but instead occurs across various tissues [39]. The elimination half-life of oral and subcutaneous semaglutide is approximately one week. The metabolites are excreted through urine and feces; approximately 3% of the drug is excreted unchanged in urine [16,39].

Adverse Effects of Semaglutide

Gastrointestinal adverse effects: nausea, vomiting, abdominal pain, and altered bowel habits are reported in 20% to 40% of patients receiving semaglutide. These adverse effects tend to be dose-dependent and likely stem from the delayed gastric emptying related to GLP-1 agonists [40,41]. Gastrointestinal intolerance is considered the most common reason for treatment cessation in patients receiving GLP-1 agonists [42]. Tolerance could be improved by initiating semaglutide at a lower dose followed by gradual up-titration [16]. Studies have raised concerns over the increased risk of pulmonary aspiration with the use of semaglutide, especially in the setting of anesthesia or sedation [43]. However, large-scale studies have failed to demonstrate a significant association between the use of GLP-1 agonists and postoperative aspiration pneumonia [44].

Pancreaticobiliary adverse effects like acute pancreatitis, cholelithiasis, and cholecystitis have been reported sporadically with semaglutide therapy. The exact mechanism is unclear; postulated mechanisms include increased cellular proliferation, suppression of cholecystokinin, and reduced drainage/stasis [45-47].

Hypoglycemia is yet another potential adverse effect. The incidence increases when used in combination with other antidiabetic medications or in the setting of persistent nausea and vomiting [16]. Furthermore, dehydration secondary to gastrointestinal intolerance could lead to pre-renal acute kidney injury or even acute tubular necrosis. Allergic reactions have been reported to GLP-1 agonists [48].

Thyroid C cell tumors: Rodent studies using GLP-1 agonists reported a higher risk of thyroid C cell hyperplasia, medullary adenoma, and carcinoma. This was explained mainly by the presence of GLP-1 receptors in rodent thyroid C cells [49]. In contrast, human thyroid parafollicular cells express minimal GLP-1 receptors [50]. The SUSTAIN trials failed to identify any cases of thyroid C-cell tumors in patients receiving semaglutide [51]. A recent systematic review by Feier et al. also reported a negligible risk of thyroid cancer with semaglutide [41]. Semaglutide continues to bear the US FDA black box warning for the potential risk of thyroid C‐cell tumors [52]. Semaglutide is contraindicated in patients with a personal or family history of medullary thyroid cancer and multiple endocrine neoplasia syndrome type 2 [40].

The adverse effects of GLP-1 agonists, including semaglutide, are summarized in Table 3.

**Table 3:** Adverse effects of GLP-1 agonists Sources: [16, 40] GLP-1: glucagon-like peptide-1; MTC: medullary thyroid carcinoma; MEN-2: multiple endocrine neoplasia type 2

Table 3: Adverse effects of GLP-1 agonists		
Adverse effects	Frequency	Mechanism / Remarks
Gastrointestinal: Nausea, vomiting, bloating, diarrhea, constipation, abdominal pain, dyspepsia	Very common (up to 50%)	Dose-dependent delayed gastric emptying and central satiety effects; usually attenuate over time
Metabolic: Decreased appetite, weight loss	Very common	Central appetite suppression (hypothalamic pathways)
Hepatobiliary: Cholelithiasis, cholecystitis	Occasional (1–3%)	Rapid weight loss increases biliary stasis and gallstone formation
Pancreatic: Pancreatitis (rare), elevated lipase/amylase	Rare (<1%)	Unclear causality; monitor if severe abdominal pain occurs
Cardiovascular: Mild tachycardia, possible hypotension	Occasional	Secondary to volume depletion or autonomic modulation
Renal: Acute kidney injury	Rare	Precipitated by volume depletion secondary to dehydration from vomiting/diarrhea/poor intake: reversible on drug discontinuation
Endocrine: Hypoglycemia	Occasional	Mostly with concomitant sulfonylureas or insulin due to synergistic effects
Neuropsychiatric: Headache, dizziness, fatigue	Common	Secondary to hypoglycemia or central action
Injection Site Reactions: Erythema, pruritus, nodules	Common (especially with exenatide, dulaglutide)	Local immune or irritative reaction
Allergic/Hypersensitivity: Rash, urticaria, anaphylaxis	Rare	IgE-mediated response
Endocrine/Neoplastic: C-cell hyperplasia, medullary thyroid carcinoma	Most date from rodent studies, theoretical risk in humans	Contraindicated in patients with personal/family history of MTC or MEN-2
Pregnancy: Fetal mortality, morphological changes, low maternal and fetal weight	Most date from rodent studies, with limited human data	Avoid during pregnancy. Recommend stopping at least two months before a planned pregnancy. Contraception is recommended for the childbearing age group

Special Population

Pregnancy: Animal studies have reported increased fetal mortality and reduced maternal and fetal weight, as well as morphological changes in the fetus, following maternal exposure to GLP-1 agonists [53]. However, literature on human maternal exposure to semaglutide is less concerning to date. In a retrospective study by Morton et al., where 12 mothers were exposed to semaglutide during the first trimester, only one fetus had anomalies. In the case of anomalies, the mother had poorly controlled DM with glycated hemoglobin of 12.5% and concomitant hypertension [54]. Multiple case reports have also reported uncomplicated fetal outcomes following exposure to GLP-1 agonists [55]. However, as of October 2025, specific to Wegovy®, the US FDA recommends cessation of the agent if pregnancy is recognized [56]. Females and males in the reproductive age group: The effect of semaglutide on human fertility is not known. The US FDA recommends Wegovy® be discontinued at least two months prior to planned pregnancy [56]. The College of Sexual and Reproductive Healthcare (CoSRH), United Kingdom, advises the use of contraception while on GLP-1 analogs [57]. The choice of contraception is an area of concern in view of possible drug interactions and a higher incidence of gastroparesis, nausea, and vomiting with GLP-1 agonists. To date, only tirzepatide is linked with poor efficacy of oral contraceptives. Accordingly, the CoSRH recommends non-oral contraceptives or additional barrier methods in patients of tirzepatide. No additional methods are recommended for semaglutide users by the society [57]. However, in the case of patients with persistent nausea or vomiting, non-oral methods or barrier methods should be adopted [58]. 

Lactation: There is limited information on the impact of semaglutide on breastfeeding. Diab et al. failed to demonstrate measurable amounts in breast milk of patients receiving up to 1 mg of subcutaneously administered semaglutide [59]. Oral semaglutide, Rybelsus®, is, however, not recommended in breastfeeding due to the presence of salcaprozate sodium, an absorption enhancer, which is secreted in breast milk and accumulates in infants [60].

Pediatrics: Semaglutide, Wegovy®, if approved, may be used for the treatment of obesity in children above the age of 12 years [56].

Renal or hepatic dysfunction: No dose adjustment for Wegovy® is recommended in patients with hepatic or renal dysfunction [38, 56].

## Conclusions

Semaglutide, initially developed for the treatment of diabetes and obesity, has now emerged as a promising therapeutic option for MASH, addressing a critical unmet need in hepatology. Its approval for non-cirrhotic MASH with fibrosis not only broadens the clinical scope of GLP-1 receptor agonists but also marks a paradigm shift toward metabolic-targeted therapies in liver disease.
